# System Design in CO_2_ Electrolysis: Integrating Value-Added Anode Reactions with Cathodic Reduction

**DOI:** 10.3390/molecules30224485

**Published:** 2025-11-20

**Authors:** Yuehui Zhai, Chong Wang, Zheng Chen

**Affiliations:** 1 School of Material and Energy, University of Electronic Science and Technology of China, Chengdu 610054, China; zhaiyuehui@uestc.edu.cn; 2 Key Laboratory of High-Performance Plastics, Ministry of Education, National and Local Joint Engineering Laboratory for Synthesis Technology of High-Performance Polymers, College of Chemistry, Jilin University, Changchun 130012, China

**Keywords:** paired electrolysis, CO_2_ electrolysis, value-added anodic reactions

## Abstract

Paired electrolysis represents a paradigm shift in overcoming the energy intensity of conventional CO_2_ electrolysis. By supplanting the oxygen evolution reaction with value-added anodic oxidations, this technology simultaneously slashes energy demands and diversifies output. This review critically synthesizes design principles for pairing these anodic reactions with two key cathode pathways, namely CO_2_ electroreduction and electrocarboxylation, supported by an analysis of pioneering systems from the past three years. We further explore advanced reactor designs for co-production, and the pivotal role of operando characterization and machine learning in unraveling complex mechanisms. We conclude with a perspective on the key challenges and pathways to industrial adoption, aiming to bridge fundamental advances with practical implementation.

## 1. Introduction

The rapid expansion of renewable electricity generation is challenged by the inherent intermittency of sources like wind, hydro-electricity, and solar power [1,2,3,4,5]. Spatiotemporal mismatches between supply and demand lead to substantial curtailment of renewable resources. While conventional storage technologies—such as pumped storage, flywheels, batteries, and compressed air—offer partial solutions, they are constrained by limited energy efficiency and high cost [6,7,8]. The direct conversion of surplus electricity into value-added chemicals, such as ammonia, presents an attractive alternative [9,10,11,12]. Electrosynthesis, which stores electrical energy in the form of chemical bonds, enables not only the storage of renewable energy but also the production of valuable fuels and chemical feedstocks [13,14,15,16].

Driven by global carbon-neutrality goals, the conversion of CO_2_ into value-added organic chemicals has become a central research pursuit [17,18,19,20]. The thermodynamic stability of CO_2_ poses a fundamental kinetic barrier, necessitating high energy inputs in conventional thermocatalytic processes that also suffer from limited efficiency and poor selectivity. Electrochemical conversion, in contrast, facilitates CO_2_ activation under mild conditions (typically temperature < 80 °C) and permits precise control over the formation of key bonds (e.g., C–C, C–O, C–N), enabling cleaner reaction pathways and tunable product distribution [21,22,23,24,25,26,27,28,29]. Furthermore, its compatibility with intermittent renewable sources allows for unstable electricity to be stored in the form of chemicals, simultaneously advancing energy storage and CO_2_ utilization.

To date, CO_2_ electrolysis has been demonstrated for a diverse range of products, from fuels like ethylene and ethanol to higher-value chemicals such as acetic acid and n-propanol, underscoring its potential to decarbonize the chemical industry. However, research has historically centered on the cathodic reduction reaction, whereas the anodic process—typically the low-value oxygen evolution reaction (OER)—has been largely neglected, limiting the overall energy efficiency and economic viability [30,31]. Paired electrolysis (also known as coupling electrolysis) has emerged as a powerful strategy to overcome these limitations [32,33,34,35,36]. By designing both half-reactions rationally, it enables the concurrent production of valuable products at the anode and cathode, significantly boosting the process’s energy efficiency, atom economy, and space-time yield.

The economic viability of CO_2_ electrolysis is increasingly critically dependent on its integration with value-added anodic reactions. Drawing on key studies from the past three years, this review outlines design principles for emerging systems, analyzes central scientific and technological challenges, and explores strategies for product diversification and pathway optimization. We aim to provide a critical framework for advancing sustainable carbon utilization technologies.

## 2. Paired Electrolysis: Fundamentals and Principles

Paired electrolysis exploits a fundamental principle of electrochemical processes: the anodic and cathodic reactions are inherently paired, and synchronized within a single cell, despite their spatial separation [37]. This concept was first systematically formalized by Baizer [38] in 1976, who posited that viable paired electrolysis should satisfy at least one of the following criteria:

1. The product of the counter-electrode reaction can be recovered and repurposed as feedstock for subsequent synthesis;
2. The cathodic and anodic processes cooperatively participate in constructing the same target molecule;
3. Each electrode generates products with independent practical value.

**Figure 1:**
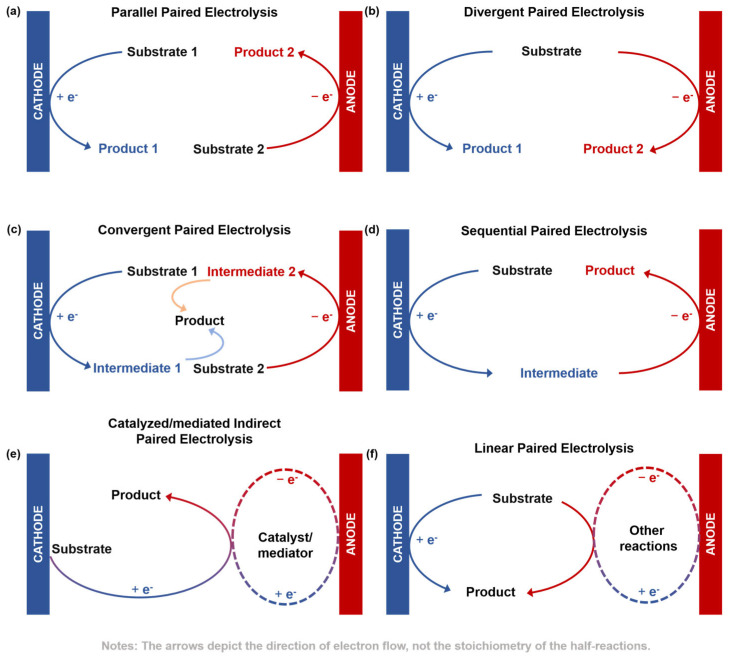
Schematics of paired electrolysis configurations. (**a**) Parallel, (**b**) divergent, (**c**) convergent, (**d**) sequential, (**e**) catalyzed/mediated indirect, and (**f**) linear.

Guided by these principles, the strategy of systematically integrating both half-reactions to maximize economic and atom efficiency has given rise to several distinct paradigms. Based on the relationship between reaction pathways and products [39,40,41,42], paired electrolysis can be categorized as follows:

1. Parallel paired electrolysis: Two different substrates are independently transformed at the cathode and anode into valuable products (Figure 1a);
2. Divergent paired electrolysis: The same substrate undergoes different reaction path-ways at two electrodes, yielding two distinct valuable products (Figure 1b);
3. Convergent paired electrolysis: Reactive intermediates (e.g., radicals or ions) generated at the cathode and anode diffuse into the solution and couple to form a single target molecule (Figure 1c);
4. Sequential paired electrolysis: A substrate undergoes sequential electron-transfer reactions, first at one electrode and then at the other, to form a single product (Figure 1d);
5. Catalyzed/mediated indirect paired electrolysis: The electrode reactions indirectly drive the target reaction by regenerating a catalyst/mediator in its active oxidation or reduction state (Figure 1e);
6. Linear paired electrolysis: A single starting material is transformed into a single target product at both electrodes. This is achieved through direct or indirect electron transfer at one electrode, while the counter electrode reaction, typically consuming a sacrificial starting material, provides a reactive species that drives the transformation toward the same product (Figure 1f).

The paired electrolysis process is driven by an externally applied cell voltage (V). This voltage must be sufficient to overcome the total cell voltage requirement, which comprises the theoretical decomposition potential (E), the ohmic drop (IR), and the anodic and cathodic overpotentials (η_a_ and η_c_). Each of these contributions follows established physicochemical principles. E is defined by the Nernst equation and evolves dynamically with the reaction progress. *IR* scales with the operating current (I), and, alongside Faraday’s law, constrains the theoretical maximum yield and rate. Finally, the relation between overpotential (η) and current density (j) is dictated by electrode kinetics; under charge transfer control, it is described by the Butler–Volmer equation [43] (which simplifies to the Tafel equation at high overpotentials).

Constant-current operation, the most common mode, inherently synchronizes the two half-reactions, maintaining this charge balance through dynamic potential adjustments [44]. While this synchronization provides considerable operational flexibility, it imposes a stringent requirement for precise reaction selectivity at each electrode. Therefore, advancing paired electrolysis towards practical application demands the co-optimization of both half-cells. Progress in this endeavor is quantified by key performance metrics that bridge fundamental research and industrial application, such as Faradaic efficiency, energy efficiency, space-time yield, and long-term stability (summarized in Table 1) [29,45,46,47]. These metrics provide a framework for assessing progress from fundamental validation toward industrial feasibility.

Notably, paired electrolysis often exhibits hybrid characteristics in systems such as tandem electrosynthesis and even in some reactions categorized under linear paired electrolysis. This is particularly evident in complex CO_2_-coupled systems. Therefore, our subsequent case analysis will transcend simplistic categorization, aiming instead to deconvolute the underlying—and often multidimensional—pairing logic.

## 3. Paired Electrolysis: Engineering Synergy Across Electrolyzers, Electrodes, and Electrolytes

The performance of paired electrolysis is fundamentally governed by the kinetic matching of its two half-reactions. Advancing beyond the conventional, isolated focus on electrocatalyst design, achieving this match demands integrated optimization across multiple scales: from the intrinsic activity of catalysts and the composition of the electrolyte, through the engineered electrode interface, to the macroscopic mass transport within the reactor. Critically, the configuration of the reactor predefines its intrinsic mass-transfer paradigm, thereby establishing the fundamental boundaries of its performance. Consequently, key performance metrics, such as total/partial current density and FE, can only be meaningfully compared across different paired electrolysis systems when evaluated under identical reactor configurations, providing a foundational basis for the fair screening of catalytic materials and reaction pathways. However, translating laboratory breakthroughs into scalable industrial processes necessitates the co-engineering of electrodes, electrolytes, and electrolyzers to actively manage these cross-scale couplings [29,48,49,50,51,52,53,54,55].

### 3.1. Configuring Electrolyzers for Kinetic and Mass Transport Synergy

The choice of reactor configuration defines the fundamental operating window, creating a technological trajectory from fundamental discovery to industrial implementation. Each configuration corresponds to a dominant mass-transfer mechanism, which in turn dictates the upper limit of the sustainable reaction rate and its suitable application scenarios. The five primary types are:

1. Beaker-type cell: The workhorse for initial reaction feasibility screening. Its simple, single-compartment design minimizes cost and internal resistance but necessitates exceptional selectivity at both electrodes to prevent cross-talk between reactants and products; its mass transport relies primarily on slow natural convection and diffusion, strictly limiting its performance ceiling.
2. H-type cell: Features a physical separator (e.g., an ion-exchange membrane) for compartmentalized studies, essential for mechanistic investigation. However, stagnant electrolytes and large electrode spacing impose severe mass transport limitations, restricting its use to low current densities. Despite its performance being incomparable to high-throughput systems, the H-cell is crucial under controlled mass-transfer conditions for assessing the intrinsic activity and selectivity of catalysts.
3. Microfluidic electrolyzer: Employs co-laminar flow in a narrow channel (<1 mm) without a membrane, enabling enhanced mass transport and continuous operation. The key drawback is significant product crossover, which leads to parasitic reactions and low Faradaic efficiency, as key intermediates can be swept away by the flow. Nevertheless, its highly controllable laminar flow characteristics allow for precise tuning of mass transfer, offering a unique platform for mechanistic studies.
4. Flow cell: A paradigm for high-rate synthesis. It integrates a membrane into a three-chamber architecture, striking a critical balance between suppressing crossover and enabling efficient ion transport. When coupled with porous gas diffusion electrodes, it can sustain industrially relevant current densities, benefiting from the extreme mass transport enabled by forced convection, albeit at the cost of increased system complexity and challenges like electrode flooding.
5. Zero-gap electrolyzer: The state-of-the-art for minimizing ohmic losses. It uses a membrane electrode assembly (MEA) to eliminate the inter-electrode gap. The membrane also defines distinct chemical microenvironments, making it ideal for gas-phase reactions. Persistent challenges include managing steep pH and water gradients, as well as the complexity of MEA fabrication. Its mass transport efficiency ranks at the top among various configurations, making it the preferred platform for high-performance system comparison and assessment of industrialization potential.

Integral to most of these advanced configurations is the separator membrane, which demarcates the cathode and anode compartments. It serves the dual function of enabling directional ion migration to complete the circuit while preventing the crossover of reactants and products, thereby preserving the distinct microenvironments essential for each half-reaction and ensuring stable operation. The choice of membrane is a system-defining decision, primarily among cation exchange membranes (CEMs), anion exchange membranes (AEMs), and bipolar membranes (BPMs). The CO_2_ electrolysis system serves as a critical case study to illustrate this dilemma. AEMs, which conduct hydroxide or carbonate ions, foster an alkaline cathode microenvironment conducive to C-C coupling. Yet, this very alkalinity accelerates carbonate formation via the reaction between CO_2_ and OH^−^, leading to significant reactant loss and diminished energy efficiency. In contrast, CEMs effectively suppress carbonate formation by transporting protons, but this action acidifies the cathode, intensifying the competing hydrogen evolution reaction (HER) and impairing CO_2_ reduction selectivity. BPMs emerge as a compelling solution by dissociating water under reverse bias, maintaining an alkaline cathode and an acidic anode, thereby simultaneously mitigating carbonate formation and product crossover. However, the substantial overpotential required for water dissociation and the associated ohmic losses currently present a major hurdle for the energy efficiency of BPM-based systems, highlighting a key area for future material innovation.

### 3.2. Electrode Design: Multi-Scale Engineering from Atomic Activity to System Integration

Beyond the electrolyzer configuration itself, the electrode’s function has fundamentally evolved from a passive catalyst support to a functional interface that actively constructs and dynamically manages the reactive microenvironment. The central challenge lies in orchestrating electron transfer, mass transport, and catalytic conversion across atomic, microscopic, and macroscopic scales to simultaneously maximize the activity, selectivity, and stability of both the anodic and cathodic reactions. The macroscopic architecture of an electrode is predominantly dictated by its dominant mass transport mechanism, and its ongoing evolution is driven by the imperative to overcome limitations in active site accessibility, mass transfer rates, and ultimate current density.

#### 3.2.1. Electrode Configurations: Architectures Defined by Mass Transport

The performance ceiling of an electrochemical process is often set by mass transport. Electrode design, therefore, begins with selecting a configuration that aligns with the physical state (gaseous or dissolved) of the primary reactant.

- Conventional two-dimensional (2D) planar electrodes (e.g., metal foils, glassy carbon): Serve as foundational model systems for fundamental electrochemical research. Operating under mass-transport-limited liquid-phase diffusion, they typically sustain current densities below 100 mA·cm^−2^. While unsuitable for high-throughput synthesis, their value resides in providing a well-defined platform with minimal confounding factors for assessing intrinsic catalyst activity and elucidating reaction mechanisms.
- Three-dimensional (3D) porous electrodes (e.g., foams, felts, meshes): Offer superior performance for reactions involving dissolved reactants, such as organic electrosynthesis or metal ion reduction. Their design centers on constructing an interconnected, hierarchical pore network to synergistically achieve a high specific surface area for catalyst loading and efficient electrolyte permeation for enhanced mass transfer.
- Gas diffusion electrodes (GDEs): Are the cornerstone of high-rate gaseous reactant conversion (e.g., CO_2_, CO, O_2_ reduction). They are engineered to establish a stable gas–liquid–solid three-phase interface, enabling operation at industrially relevant current densities exceeding 1 A·cm^−2^. Their design lies in maintaining this interface’s dynamic stability under high-rate conditions through meticulous gradational control from a macroporous, hydrophobic gas diffusion layer (GDL) to a mesoporous, hydrophilic catalyst layer (CL).
- Surface-engineered nanostructured electrodes (e.g., nanowire arrays, nanotube forests): Extend the design paradigm to the nanoscale. They aim to provide directional charge transport pathways and, through precise interface engineering, actively tailor the local chemical microenvironment (e.g., pH, reactant concentration). This creates opportunities to explore and optimize demanding reaction pathways with high energy barriers that are inaccessible to conventional morphologies.

#### 3.2.2. Overarching Design Principles: A Multi-Scale Philosophy

Transcending specific configurations, superior electrode design adheres to a set of integrated, multi-scale principles:

1. Atomic-scale electronic structure modulation: The foundational principle. Strategies such as inducing strain, engineering coordination environments (e.g., M-N_4_ sites), and incorporating dopants are employed to optimize the adsorption energy of key reaction intermediates. This allows for precise steering of the reaction pathway towards desired products while suppressing competing reactions (e.g., HER). This principle applies broadly, from CO_2_ reduction (tuning Cu-based catalysts for multi-carbon products) to the selective oxidation of organic molecules.
2. Microscale mass transport and mesoscale charge transfer optimization: The electrode architecture must facilitate efficient transport highways for reactants and products. This is achieved by designing hierarchical pore structures that align with the reactant’s physical state (gas/liquid) and ensure continuous accessibility of active sites, accounting for timely product (especially gas) removal to prevent pore blocking. Concurrently, the electrode must ensure low-resistance electronic pathways to all active sites.
3. System-level chemical and mechanical compatibility: Electrodes cannot be designed in isolation. They must be co-optimized with the membrane and electrolyte to mitigate detrimental crossover processes (e.g., product oxidation, salt precipitation) and ensure long-term operational durability under harsh conditions, including extreme potentials, pH gradients, and mechanical stress.
4. Scalable manufacturing and sustainability-by-design: For meaningful industrial translation, electrode design must incorporate forward-looking considerations for scalable fabrication processes (e.g., roll-to-roll manufacturing), the use of Earth-abundant materials, and end-of-life recyclability based on green design principles, ensuring both economic and environmental viability.

### 3.3. Electrolyte Engineering: Tailoring the Ionic and Chemical Microenvironment

The electrolyte, serving as the medium for ion transport and the source of reacting species, plays a decisive role in shaping the interfacial microenvironment at both electrodes, thereby governing the activity, selectivity, and stability of the entire paired system. Its design must be holistically integrated with the choice of electrolyzer and electrode architecture.

#### 3.3.1. Cation and Anion Effects: The Ionic Helmholtz Layer

The specific identity of ions in the electrolyte directly modulates the electrochemical double layer, influencing reaction pathways beyond mere conductivity.

- Cations (e.g., Li^+^, Na^+^, K^+^, Cs^+^): Their size and hydration energy affect the electric field strength at the electrode surface. Larger, less hydrated cations (e.g., Cs^+^) can more effectively stabilize anionic reaction intermediates (e.g., CO_2_·^−^ in CO_2_RR) and lower the activation barrier for C–C coupling steps, following the Hofmeister series.
- Anions and pH regulation: The bulk and local pH, dictated by the buffering capacity of the electrolyte, is a master variable. Alkaline media (e.g., KOH) thermodynamically favor certain reactions like C–C coupling but can cause carbonate precipitation and catalyst degradation. Acidic media (e.g., H_2_SO_4_/KCl mixtures) prevent carbonate formation but require the in situ generation of a local high-pH environment at the cathode to suppress the HER. Neutral buffers (e.g., KHCO_3_) offer a compromise but with limited buffering capacity at high current densities. Anions can also specifically adsorb onto catalyst surfaces, altering the electronic structure of active sites and the adsorption energy of key intermediates.

#### 3.3.2. Solvent and Additive Engineering

The performance of an electrolyte can be radically advanced by moving beyond conventional solvents and incorporating functional additives:

- Solvent selection: Moving beyond aqueous systems, organic solvents (e.g., acetonitrile) and ionic liquids can drastically increase the solubility of non-polar reactants (e.g., CO_2_, organic substrates) and provide a different dielectric environment, opening alternative reaction pathways and selectivity.
- Functional additives: Small amounts of halide ions (Cl^−^, Br^−^, I^−^) or organic molecules can act as promoters or surface modifiers. They can selectively block sites for parasitic reactions (e.g., HER), enhance local CO_2_ concentration by modifying surface hydrophobicity, or directly participate in stabilizing critical reaction intermediates.

#### 3.3.3. System Integration and Trade-Offs

The electrolyte formulation is inextricably linked to the other cell components.

- Synergy with electrodes: The electrolyte’s wetting behavior and viscosity must be compatible with the electrode’s pore structure—flooding of GDEs must be prevented, and mass transport within 3D electrodes must be ensured.
- Compatibility with membranes: The electrolyte’s pH and composition must be compatible with the membrane’s operational stability window, and vice versa. For instance, AEMs require alkaline conditions, while CEMs are suited for acidic environments. BPMs allow for the independent optimization of anolyte and catholyte, a powerful yet complex strategy.
- Serving the paired reactions: The ultimate goal is to formulate an electrolyte (or anolyte/catholyte pair) that sustains optimal microenvironments for both the cathode and anode reactions simultaneously, balancing the needs for reactant supply, product removal, and inhibition of cross-reactions.

The pursuit of efficient paired electrolysis thus converges on a single, overarching challenge: the management of chemical and ionic gradients across multiple scales. As detailed above, the choice of electrolyzer configuration defines the macroscopic platform for reaction engineering, the design of hierarchical electrodes dictates the micro-scale transport and local environment, and the formulation of the electrolyte directly tailors the atomic-scale interface where reactions occur. Future breakthroughs will not come from the isolated optimization of any single component, but from their intentional synergy. This requires the integrated design of electrodes that are architected to function within the specific ionic environment created by the electrolyte and membrane, all housed within a reactor that facilitates the necessary mass transport. Successfully mastering this integration is the key to unlocking high-rate, selective, and energy-efficient electrochemical synthesis for a sustainable chemical industry.

## 4. Paired Electrolysis in Electrocarboxylation

Electrocarboxylation has emerged as a powerful strategy for utilizing CO_2_ as a C1 synthon in the construction of C–C and C–X (X = F, Cl, Br, O, N, S) bonds, as well as functionalization carbon-based π systems, to access high-value chemicals [25,56,57,58,59]. Early methodologies predominantly relied on sacrificial anodes (such as Al, Mg, and Zn) to manage charge balance and reaction selectivity. However, these systems were inherently constrained by cost and scalability issues due to continuous electrode consumption [60,61]. A paradigm shift has since moved the field toward non-consumable anodes, which capitalize on the anodic half-reaction to co-generate valuable oxidation products, thereby improving process economics [62,63,64,65,66,67].

**Figure 2:**
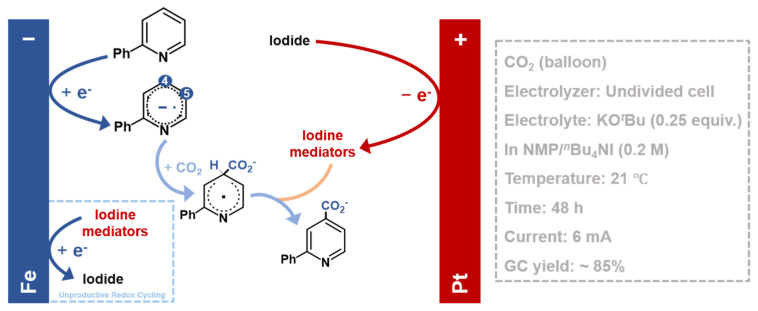
Mechanism for the iodide-mediated paired electrocarboxylation of 2-phenylpyridine. This approach enables electrocarboxylation without a sacrificial anode, based on Ref. [63].

A seminal study by Yu et al. [63] demonstrated how paired electrolysis can override intrinsic thermodynamic preferences to achieve switchable C–H electrocarboxylation of N-heteroarenes (Figure 2). In a three-necked round-bottom (a beaker-type cell) using 2-phenylpyridine, they showed that while the cathodically generated pyridine radical anion intermediate undergoes reversible C5-electrocarboxylation with CO_2_ (the thermodynamic pathway), this intermediate is reactive with iodine mediators (I_2_/I_3_^−^). These species, generated from the oxidation of iodide ions (I^−^) at the anode, diffuse to the cathode and instigate an irreversible hydrogen atom transfer (HAT) preferentially at the C4 position, a site favored by its lower C–H bond dissociation energy. This diverts the reaction pathway under Curtin-Hammett control, thereby rendering the kinetically controlled C4-electrocarboxylation dominant. Notably, iodide serves a dual role as a redox mediator and electrolyte, establishing a non-sacrificial catalytic cycle where the iodine species are reductively recycled. This work clearly underscores that pairing anodic and cathodic events is a potent strategy for unlocking novel and selective reaction pathways.

**Figure 3:**
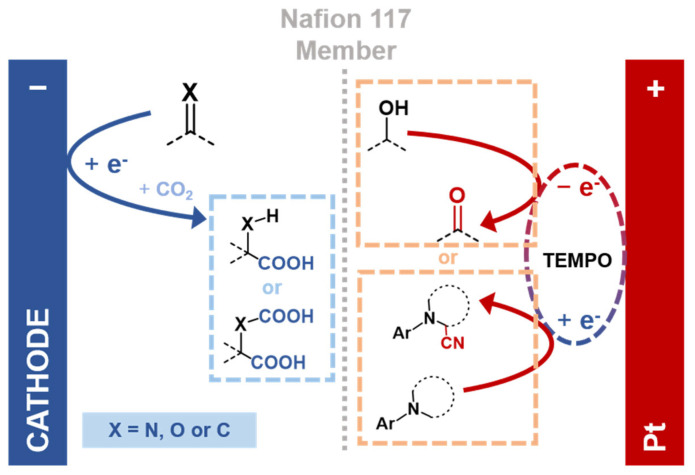
Schematic of a sacrificial-anode-free paired electrolysis producing high-value pharmaceutical intermediates via cathodic electrocarboxylation of unsaturated bonds and anodic TEMPO-mediated electro-oxidation of alcohols/amines, based on Ref. [66].

Moving beyond conventional carboxylation, Dong et al. [66] conceptualized a synergistic parallel-paired system that couples two productive half-reactions in a single electrochemical cell. Their design co-opts the cathodic electrocarboxylation of substrates like ketones and alkenes with the anodic oxidative of alcohols or amines, effectively eliminating the need for sacrificial electrodes or reagents (e.g., electrocarboxylation of 2-acetyl-6-methoxynaphthalene with oxidation of 4-methoxybenzyl alcohol) (Figure 3 and Table 2). Elucidation of the mechanisms across a wider range of substrates confirmed distinct reaction pathways. Cathodically, ketones form ketyl radical anions, while alkenes react with CO_2_·^−^ radicals for regioselective electrocarboxylation. Anodically, TEMPO-mediated oxidation bifurcates between alcohol oxidation and Shono-type cyanation of amines via iminium ions. Demonstrating broad substrate scope, the protocol was effectively applied to synthesize key intermediates for pharmaceuticals including naproxen and ibuprofen, highlighting its potential for atom- and energy-efficient synthesis.

Despite these compelling demonstrations of synthetic utility, a significant challenge persists in the field: the prevalent gap between mechanistic studies and energy-oriented analyses. While considerable insights into reactivity and yield have been gained, comprehensive reporting of key figures of merit—such as FE, cell voltage, and overall energy efficiency—remains uncommon. This omission fundamentally limits the rigorous benchmarking and cross-comparison of energy utilization across different coupled electrolysis platforms.

## 5. Valorized Anode Reactions with Electrochemical CO_2_ Reduction Reaction (CO_2_RR)

A seminal 2019 techno-economic analysis by Kenis et al. [30] identified the high energy demand of CO_2_RR as its primary economic impediment. While CO_2_RR mechanisms and catalysis have been extensively reviewed, the research paradigm is now shifting from isolated cathode optimization to integrated system design. This transition addresses two intertwined challenges. First, the anodic oxygen evolution reaction (OER), with its substantial overpotential, dominates the system’s energy consumption. Second, in the necessary alkaline/neutral media, hydroxide ions generated at the cathode react with CO_2_ to form (bi)carbonates. This parasitic process can consume over 70% of the input CO_2_, severely compromising carbon efficiency. To circumvent these limitations, strategies are increasingly focusing on pairing CO_2_RR with alternative anode reactions. These reactions—selected organic and inorganic oxidations (OOR/IOR)—are promising substitutes [67,68,69,70,71,72,73,74,75,76,77,78,79,80,81,82,83,84,85,86,87,88,89,90,91,92,93,94,95,96] due to their lower thermodynamic potentials or favorable kinetics (e.g., preferential chloride oxidation), both of which can directly lower the cell voltage. Crucially, the choice of anode reaction directly governs the system’s proton flux and local pH environment. By substituting the proton-consuming OER with OOR/IOR that generate or conserve protons, these systems can inherently suppress cathode alkalization. This co-design approach simultaneously mitigates carbonate formation and reduces energy demands, transforming the anode from an energy sink into a source of valuable products. The outcome is a concurrent enhancement of overall energy efficiency, carbon utilization, and process economics. Table 3 summarizes key anode reactions and their impact on proton flux.

### 5.1. Paired Electrolysis via OOR

**Figure 4:**
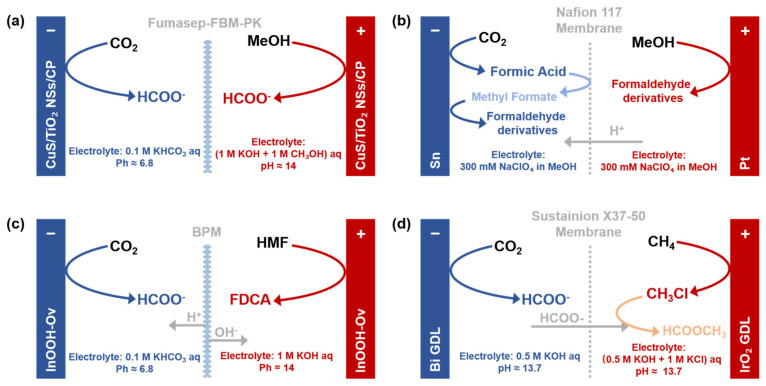
Schematic of paired CO_2_ electrolysis systems with integrated anodic oxidation of MeOH (alcohol), HMF (biomass-derived aldehyde), or CH_4_ (fuel) in H-cell (**a**–**c**) and flow cell (**d**) configurations, based on Refs. [75,79,80,86].

The paradigm of anode reactions is shifting from the energy-intensive OER toward value-added anodic transformations. A prominent example is the oxidation of alcohols, which proceeds at lower potentials than the OER to upgrade inexpensive feedstocks into valuable products such as aldehydes and carboxylic acids (or their carboxylate salts) [72,73,74,75,76,77]. Exemplifying this strategy, Zhu et al. [75] employed a CuS/TiO_2_ nanosheet heterostructure as a bifunctional catalyst for paired electrosynthesis (Figure 4a), achieving simultaneous formate production from both CO_2_RR and methanol oxidation (MOR). The system delivered a formate FE of 75.4% at the cathode (at −0.9 V vs. RHE) and nearly 100% at the anode, at a total current density of 10 mA·cm^−2^. This remarkable performance originates from the tailored interfacial design of the heterojunction: at the cathode, surface hydroxyl groups on TiO_2_ optimize the local proton supply, steering the selective conversion of CO_2_ to formate; concurrently, at the anode, an analogous interfacial structure creates a hydroxyl-rich environment that guides the highly selective oxidation of methanol to formate, replacing the energy-intensive oxygen evolution reaction. This precise synergy, underpinned by enhanced electron transfer and reaction kinetics, thereby enhances both the overall energy efficiency and process economics by co-producing a single valuable chemical at both electrodes.

The utility of anodic oxidation in valorization processes is not limited to carboxylic acids. A notable example is the work of Nam et al. [79], who reported the co-production of formaldehyde derivatives via a paired electrolysis system that couples CO_2_RR with methanol oxidation (Figure 4b). To circumvent the thermodynamic and kinetic barriers associated with the direct cathodic reduction of CO_2_ to formaldehyde, the authors devised an electrocatalytic system featuring a bio-inspired “activation–stabilization” mechanism, analogous to the Calvin cycle. In this configuration, CO_2_ undergoes a two-electron reduction to formic acid on a Sn cathode. The formic acid then undergoes rapid, proton-catalyzed esterification with methanol to yield methyl formate (MF). Subsequent electrochemical reduction in MF generates formaldehyde, which is simultaneously trapped as stable derivatives within the acidic catholyte. Crucially, the required protons are supplied in situ by the partial electro-oxidation of methanol at a Pt anode, methanol is selectively oxidized via a two-electron pathway to formaldehyde, avoiding over-oxidation to CO_2_. These protons migrate through a Nafion-117 membrane to the cathode compartment. Operating at a total current density of 10 mA cm^−2^, this synergistic process achieves high Faradaic efficiencies up to 50% for the cathodic formaldehyde derivatives and 90% for the anodic formaldehyde, establishing an atom-economical route for CO_2_ valorization.

The valorization of organic molecules via anodic extends beyond simple alcohols [80,81,82,83,84,85]. In a representative study, Hu et al. [80] developed a bifunctional InOOH-OV catalyst that achieves a FE of 92.6% for CO_2_-to-formate conversion concurrently with a 91.6% yield of FDCA from HMF oxidation (Figure 4c). Mechanistic studies reveal that oxygen vacancies induce lattice distortion and charge redistribution, which collectively stabilize the catalyst structure and optimize the adsorption of key intermediates, thereby enabling the exceptional dual activity. Capitalizing on this catalyst, the authors engineered a pH-asymmetric electrolyzer incorporating a bipolar membrane (BPM). The BPM—comprising an anion-exchange layer (AEL), a cation-exchange layer (CEL), and an interfacial layer (IL)—dissociates water under bias, enabling H^+^ migration to the cathode to sustain CO_2_RR and OH^−^ migration to the anode to facilitate HMF oxidation, thereby maintaining optimal pH environments in spatially decoupled compartments. Operating at a cell voltage of 2.27 V, the system co-produces formate at ~90% FE and FDCA at 87.5% yield, demonstrating high efficiency. This seminal work not only provides fundamental insights into the design of p-block metal oxide catalysts but also compellingly underscores the potential for the integrated electrochemical conversion of CO_2_ and biomass into valuable chemicals in a single device.

Methane, a major constituent of natural gas and a potent greenhouse gas, is gaining increasing attention. Similarly to CO_2_, methane’s high chemical inertness typically demands energy-intensive, high-temperature processes for its conversion. Pioneering work by Zheng et al. [86] established an integrated paired electrolysis system for the simultaneous conversion of CO_2_ and CH_4_, enabling the direct electrosynthesis of methyl formate (Figure 4d). This approach sets a new paradigm for the synergistic valorization of greenhouse gases. In their design, CO_2_ is reduced to formate on a Bi cathode, while CH_4_ is oxidized to CH_3_Cl via a Cl^−^-mediated pathway on an IrO_2_ anode. Crucially, the electro-generated formate migrates to the anode compartment, where it engages in an electric-field-driven nucleophilic substitution with CH_3_Cl to yield the final product. Operating at a cell voltage of ~3.6 V (vs. RHE), this integrated system achieves a peak methyl formate production rate of 1660 μmol·h^−1^·cm^−2^ and a remarkable full-cell energy efficiency of ~15.2%, substantially outperforming the ~4.2% efficiency of decoupled processes. Moreover, the system exhibits exceptional operational stability, maintaining an industrial-grade current density of 700 mA·cm^−2^ for over 8 h with an accumulated product yield of ~12,000 μmol·cm^−2^, underscoring its potential for practical application.

**Figure 5:**
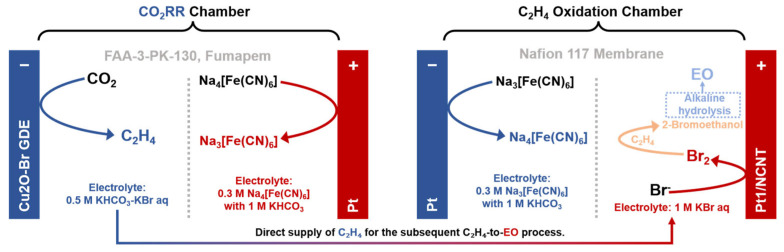
Schematic of a paired electrolysis system where a Na_4_[Fe(CN)_6_]/Na_3_[Fe(CN)_6_] redox mediator substantially lowers the system overpotential, enabling the Br-mediated, single-pass conversion of CO_2_ to EO in a tandem flow cell, based on Ref. [87].

To address inherent challenges such as reaction rate mismatch and mass-transfer limitations, tandem paired electrolysis in membrane-separated reactors has emerged as a promising solution. A representative example is the work by Zhu et al. [87] on the tandem synthesis of ethylene oxide (EO) from CO_2_ (Figure 5). In a bromine-mediated flow cell system, electrocatalytic synergy enables efficient tandem conversion of CO_2_ to EO. At the anode, in situ-generated Br_2_/HBrO over a Pt_1_/NCNT catalyst facilitates ethylene epoxidation to EO, exhibiting a FE of 92.2% at 50 mA·cm^−2^ with stable performance over 15 h. On the cathode, bromide ions stabilize Cu^+^ sites within a porous Cu_2_O electrode, achieving a 66.9% FE for CO_2_ reduction to C_2_H_4_ at 800 mA·cm^−2^ and maintaining stability for 24 h at 500 mA·cm^−2^. Crucially, replacing energy-intensive water splitting with a Na_4_[Fe(CN)_6_]/Na_3_[Fe(CN)_6_] redox mediator substantially lowers the system overpotentials, reducing the CO_2_ reduction working voltage to −1.41 V at 300 mA·cm^−2^ (a saving of ~1.55 V) and enabling C_2_H_4_ epoxidation at 2.0 V for 100 mA·cm^−2^ (a saving of ~1.18 V). The integrated tandem system achieves an overall 41.1% single-pass FE for CO_2_-to-EO conversion (with the CO_2_RR chamber operating at 800 mA·cm^−2^ and the C_2_H_4_ oxidation chamber at 25 mA·cm^−2^), underscoring its promise for energy-efficient electrosynthesis.

### 5.2. Paired Electrolysis via IOR

**Figure 6:**
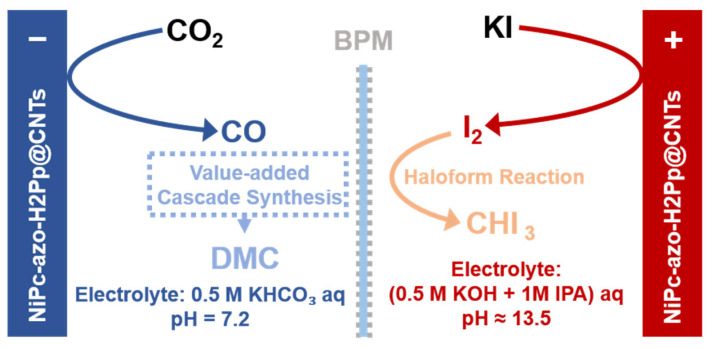
Schematic of a value-added system where paired electrolysis (CO_2_RR and IOR) is followed by a cascade reaction (CO-to-DMC) to achieve substantial energy savings, based on Ref. [88].

Replacing the OER with thermodynamically more favorable and kinetically faster oxidation reactions presents a pivotal strategy to overcome the energy consumption issue in the CO_2_RR, the oxidation of halide is a prime example of this approach [88,89,90]. Unlike the sluggish four-electron OER, halide oxidation typically follows a kinetically rapid two-electron pathway, as exemplified by the well-established chlorine evolution reaction in the chlor-alkali industry. Demonstrating this potential, Zhu et al. [88] developed a symmetric paired electrolysis system employing a bidirectional electrocatalyst (NiPc-azo-H_2_Pp@CNTs) that concurrently couples CO_2_RR with the iodide oxidation reaction (IOR) (Figure 6). This catalyst facilitates proton-coupled electron transfer to promote CO_2_-to-CO conversion at the cathode, while simultaneously driving the IOR to generate I_2_ at the anode. The electrogenerated I_2_ subsequently undergoes a spontaneous haloform reaction to produce valuable iodoform (CHI_3_), effectively replacing the energy-intensive oxygen evolution reaction (OER). This integrated CO_2_RR–IOR system requires a cell voltage of only 2.98 V at 30 mA·cm^−2^, substantially lower than the 3.74 V needed for a conventional CO_2_RR–OER system, corresponding to a ~20% reduction in energy consumption. The system operated stably for 5 h at 25 mA·cm^−2^, continuously producing high-purity CO at the cathode and delivering 168 mg of iodoform at the anode. Moreover, the CO produced was further utilized in a cascaded process to synthesize another high-value product, dimethyl carbonate (DMC), at a rate of 6.21 mmol·L^−1^·h^−1^.

**Figure 7:**
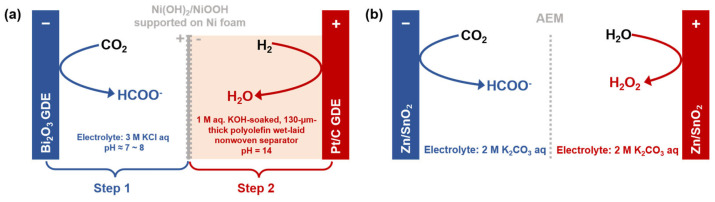
Schematics of CO_2_ electrolysis systems through paired electrolysis: (**a**) CO_2_RR–HOR coupling via a Ni(OH)_2_/NiOOH redox mediator for low-voltage formate production, and (**b**) simultaneous CO_2_RR-to-formate and H_2_O_2_ production from H_2_O oxidation, based on Refs. [91,92].

Beyond halide oxidation, the hydrogen oxidation reaction (HOR) presents an alternative strategy to reduce cell voltage by leveraging its low thermodynamic potential. Yan et al. [91] developed an integrated CO_2_ electrolysis system coupled with HOR, utilizing a Ni(OH)_2_/NiOOH redox mediator to spatially and temporally decouple the reactions (Figure 7a). The system operates in a cyclic, two-step manner: in Step 1, CO_2_ is reduced to formate on a Bi_2_O_3_ catalyst while Ni(OH)_2_ is oxidized at the anode; in Step 2, NiOOH is reduced back via HOR, releasing energy that partially offsets the input from Step 1. This mediator-assisted approach replaces the OER and mitigates issues of anode carbon loss and HOR catalyst poisoning. The system achieves a low operating voltage of 0.89 V for formate production at 50 mA·cm^−2^, with a minimal voltage decay of only 0.12 V over 100 h. When coupled with hydrogen from an alkaline or solid oxide electrolyzer, the integrated system reduces total energy consumption by up to 42% compared to conventional CO_2_RR–OER systems. This work demonstrates a feasible path for coupling CO_2_RR with grid-scale energy storage and provides foundational insights for designing highly efficient, integrated energy systems.

In aqueous electrolytes, the two-electron water oxidation to hydrogen peroxide (H_2_O_2_) offers an attractive anodic alternative [92,93,94]. You et al. [92] reported an innovative coupled electrolysis system driven by a single bifunctional catalyst (Zn/SnO_2_ nanodots) for the simultaneous CO_2_RR to formate with H_2_O_2_ production (Figure 7b). Their study revealed that Zn doping plays a dual role: at the anode, it tunes the electronic structure to favor the coupling of adsorbed *OH intermediates for selective H_2_O_2_ generation, while suppressing the competing oxygen evolution reaction; at the cathode, it stabilizes the *OCHO intermediate, steering the CO_2_ reduction toward formate with high selectivity. The full cell achieved a current density of 150 mA·cm^−2^ at 4.2 V, affording FEs of 80.6% for H_2_O_2_ and 92.2% for formate. The system maintained stable operation for over 60 h without performance decay (FE_H2O2_ ≈ 72.8%, FE_formate_ ≈ 85.7%).

**Figure 8:**
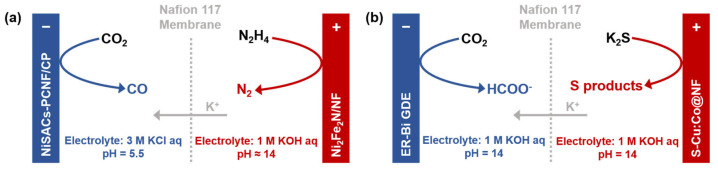
Schematics of paired electrolysis systems for (**a**) CO_2_RR and hydrazine oxidation to N_2_, and (**b**) CO_2_RR and sulfide oxidation to sulfur, in flow cells featuring K^+^ migration through a CEM for charge balance, based on Refs. [95,96].

Finally, the integration of waste valorization with CO_2_RR presents a sustainable pathway offering dual environmental benefits [95,96]. Hydrazine, a high-energy-density rocket propellant, poses significant ecological threats through industrial use and residual fuel contamination. Exemplifying this strategy, Wen et al. [95] reported a sophisticated paired-electrolysis system that couples CO_2_RR with the hydrazine oxidation reaction (HzOR) (Figure 8a). This design simultaneously addresses two challenges: valorizing CO_2_ and detoxifying an environmental pollutant into benign N_2_. Configured in a membrane-separated flow cell, their system employed a nickel single-atom catalyst for efficient CO_2_-to-CO conversion at the cathode and a Ni_2_Fe_2_N anode that oxidized hydrazine at a profoundly low potential. The synergy between these reactions is underscored by the full cell’s ultra-low operating voltage of 0.45 V to achieve an industrial current density of 100 mA·cm^−2^. Under these conditions, the system sustained a FE_CO_ of >80% and a full-cell energy efficiency of >40%. Furthermore, when assessed for long-term stability at a lower cell voltage of 0.4 V, the system maintained a steady current density of ~40 mA·cm^−2^ with consistent selectivity over 15 h of continuous operation.

In a similar vein, sulfides (e.g., H_2_S or S^2−^), another class of toxic pollutants, can be efficiently managed through paired electrolysis. As demonstrated by Liu et al. [96], a system coupling sulfide oxidation reaction (SOR) with CO_2_RR was developed (Figure 8b). It employs a S-Cu:Co@NF anode to convert HS^−^ into value-added polysulfides (S_x_^2−^) in an alkaline electrolyte, while an ER-Bi gas diffusion electrode cathode selectively reduces CO_2_ to formate. Theoretical calculations reveal that Cu and S co-doping modulates the electronic structure of the Co(OH)_2_ catalyst, optimizing the adsorption free energy of key sulfur intermediates and thus accelerating SOR kinetics. At an industrial current density of 100 mA·cm^−2^, the cell voltage is only 2.10 V, with low anodic and cathodic overpotentials of 0.24 V and 0.48 V, respectively. Both half-reactions achieve FEs exceeding 90%, reducing the overall energy consumption by approximately 40% compared to conventional CO_2_RR-OER systems. When integrated with commercial photovoltaics, the system operates stably for over 6 h at 100 mA·cm^−2^, achieving a solar-to-chemical energy conversion efficiency of 5.8% (major product: formate). These works exemplify a strategic paradigm that not only slashes the energy cost of CO_2_ electrolysis but also opens a sustainable avenue for synergistic waste management and chemical production.

## 6. In Situ/Operando Characterizations and Machine Learning of CO_2_ Electrolysis

Understanding the complex dynamics of CO_2_ electrolysis, multi-technique in situ/operando characterizations have emerged as a pivotal strategy for deciphering interfacial adsorption, intermediate evolution, and product selectivity [97,98,99]. A representative study by Chen et al. [97] correlated in situ X-ray absorption spectroscopy (XAS), transmission electron microscopy (TEM), and X-ray diffraction (XRD) to investigate a single-atom copper catalyst during CO_2_RR. Their work revealed intrinsic correlations among dynamic atomic configuration, chemical state transformation, and catalyst’s surface Coulomb effects, establishing a clear structure-performance relationship. Extending these powerful probes to paired CO_2_ electrolysis systems is essential. This approach enables the simultaneous and correlated probing of dynamic evolution at both the anode and cathode interfaces, which is essential for revealing the microscopic essence of synergistic enhancement and avoiding mutually inhibitory side reactions. This advancement represents a critical leap for the field, moving from phenomenological description toward rational design guided by mechanistic understanding.

Beyond capturing dynamic structural evolution, optimizing the CO_2_ electrolysis process is challenging, as it is governed by the non-linear coupling of multiple parameters, including operating potential, electrode structure, electrolyte composition, and electrolyzer configuration. The complex interactions among these parameters pose a high-dimensional challenge for system optimization. Machine learning (ML) is uniquely suited to address this challenge by establishing quantitative “condition-performance” mappings [100,101,102]. For instance, Cuenya et al. [101] integrated in situ XAS with both unsupervised and supervised ML, successfully resolving the dynamic coordination structures of multiple metal sites and their interactions with key intermediates in transition metal-nitrogen-doped carbon materials under working conditions. This provided atomic-level insights into the dynamic process of CO_2_RR. Although ML has demonstrated significant potential in material characterization and reaction mechanism studies within CO_2_ electrolysis, its application in the co-optimization of anodic and cathodic reactions to enhance overall system efficiency remains a nascent and critical frontier for future research.

## 7. Summarization and Prospect

Coupling anodic reactions in CO_2_ electrolysis represents a transformative strategy for decarbonizing chemical synthesis, offering substantial energy savings and a broader portfolio of products. However, bridging the gap between laboratory promise and industrial deployment requires addressing multi-faceted challenges. Key bottlenecks span technical, economic, and scientific realms. Technically, the absence of unified metrics hinders objective comparison, while the scarcity of scalable reactor designs presents another major hurdle. Economically, prevailing assessments overlook synergies such as paired electrolysis and market volatilities. Scientifically, the complex reaction networks remain underexplored. This challenge is compounded by the nascent state of research, which has yet to fully harness tools like machine learning. Overcoming these hurdles demands a fundamental shift from isolated reaction optimization to a holistic, system-level understanding of reaction synergies. This paradigm shift must be coupled with parallel innovations in electrolyzer architecture and system integration. By embedding the unique economics of paired processes into robust techno-economic frameworks and fostering cross-disciplinary convergence, this promising technology can transition from a compelling concept to a commercially viable cornerstone of a sustainable carbon cycle.

## Figures and Tables

**Table 1:** Common key metrics and design considerations for paired electrolysis systems.

Name	Definition *	Role in System Design *
(Total) Current Density (j)	The electric current flowing per unit geometric area (or electrochemical active surface area) of the electrode.	Determines the reaction rate and reactor’s scale; a primary driver of the capital expenditure.
Faradaic Efficiency (FE)	The fraction of the total electric charge used to produce a specific desired product.	Evaluates the selectivity of each half-reaction; the product of the FE at both electrodes defines the upper limit of the system’s atom economy and revenue potential.
Cell Voltage (V_cell_)	The total voltage applied between the anode and cathode required to drive the stable operation of the entire electrolyzer.	Directly determines the system’s energy consumption; the primary determinant of operating expenses.
Energy efficiency (EE)	The ratio of the total chemical energy stored in the products (anodic and cathodic) to the total electrical energy input into the system.	A comprehensive metric of overall energy utilization, highlighting the synergy from co-producing valuable products at both electrodes.
Electrode Stability	The rate of performance decay over time for a single electrode under operational conditions.	Ensures the longevity operational reliability of each half-cell; a prerequisite for system-level stability.
System Stability	The duration over which the entire paired electrolysis system maintains its target performance	The critical metric for commercial viability, assessing the compatibility and mutual degradation of all integrated components under paired conditions; often the key gap between lab-scale demonstration and industrial application.
Space-Time Yield (STY)	The mass of product produced per unit time per unit reactor volume.	Measures the productivity and compactness of the reactor; key for process intensification.
Product Concentration/Putative	The concentration of the target product in the outlet electrolyte stream, or the purity of a gaseous product.	Directly determines the energy and cost intensity of downstream separation and purification.
Techno-economic Analysis (TEA)	A systematic framework for quantifying the technical and economic feasibility of a technology.	Translates the technical synergies of paired electrolysis into a clear assessment of its commercial viability.

* The performance of all metrics is highly dependent on operating conditions (e.g., current density, electrochemical active surface or geometric area of electrolyte, catalyst, temperature, pH). For meaningful comparison across systems, the specific testing conditions are required to be explicitly reported.

**Table 2:** Electrolysis under experimental conditions in Dong et al.

Cathode System	Cathode Substrate	Cathode Electrode/ Electrolyte	Cathode Product and FE*	Cathode Potential	Paired Anode Substrate	Anode Electrode/ Electrolyte	Anode Product and FE *	Anode Potential	Full Cell Voltage
Ketone	2-Acetyl-6-methoxynaphthalene	Electrode: Pb sheet Electrolyte: Bu_4_NClO_4_ (0.1 M) Solvent: MeCN	2-Aryl lactic acid FE: 95%	~−2.23 V vs. Fc/Fc^+^	4-Methoxybenzyl alcohol	Electrode: Pt sheet Electrolyte: Bu_4_NBr (0.1 M) Solvent: MeCN Additives: TEMPO, 2,6-lutidine	4-Methoxybenzaldehyde FE: 73%	~0.242 V vs. Fc/Fc^+^	~10.55 V

* FE denotes the coulombic yield for each half-reaction, with the cathodic contribution adjusted according to its stoichiometric electron demand. Electrode potentials were measured against Ag/AgI reference electrodes and are referenced to the Fc/Fc^+^ couple. Full cell voltage was recorded directly from the power supply. Standard conditions employed a constant current of 20 mA, a temperature of 5 °C, and atmospheric-pressure CO_2_ introduced via bubbling into the cathodic compartment.

**Table 3:** Anode reactions for CO_2_ electrolysis and their impact on the local environment.

Cell Type	Anodic Reaction	Type and Role of Membrane	Proton Flux & Charge Compensation	Ref.
H-cell (no GDE)	CH_3_OH + 4OH^−^ → HCOO^−^ + 3H_2_O + 3e^−^	BPM interface enables water dissociation to balance charge and prevent pH crossover, critical for stabilizing environments in paired electrolysis.	Cathodic H^+^ modulation via TiO_2_ nanosheets; anodic H^+^ effects not addressed.	[75]
H-cell (no GDE)	CH_3_OH → HCHO + 2H^+^ + 2e^−^	CEM maintains the directional migration of H^+^ to balance the charge and prevent crossover between the electrodes.	Cathodic H^+^ modulation directly determines by the anode reaction	[79]
H-cell (no GDE)	HMF + 6OH^−^ → FDCA + 4H_2_O + 6e^−^	BPM, water dissociation at the interface; supplies H^+^ and OH^−^ to balance the charge and prevent crossover between the electrodes.	None H^+^ flux from the anode; cathode H^+^ modulation directly determines by the BPM.	[80]
Flow cell (with GDE)	CH_4_ + Cl^−^ → CH_3_Cl + H^+^ + 2e^−^	AEM, guided migration of HCOO^−^ from cathode to anode and prevent crossover between the electrodes.	None H^+^ flux from the an-ode; H^+^ generated at the anode undergoes immediate neutralization by the abundant OH^−^ in anolyte.	[86]
Tandem flow cell system (with GDE)	[Fe(CN)_6_]^4−^ → [Fe(CN)_6_]^3−^ + e^−^ (CO_2_RR chamber) 2Br^−^ → Br_2_ + 2e^−^ (C_2_H_4_ oxidation chamber)	AEM (CO_2_RR chamber), selective ion transport (predominantly anions) for environmental charge balance and prevent crossover between the electrodes.	None H^+^ flux from the an-ode in CO_2_RR chamber.	[87]
Flow cell (with GDE)	3I^−^ → I_3_^−^ + 2e^−^	BPM, water dissociation at the interface; supplies H^+^ and OH^−^ to balance the charge and prevent crossover between the electrodes.	Negligible H^+^ flux from the anode; cathode H^+^ modulation directly determines by the BPM.	[88]
Flow cell (with GDE)	Ni(OH)_2_ + OH^−^ → NiOOH + H_2_O + e^−^ (Step 1) H_2_ + 2OH^−^ → 2H_2_O + 2e^−^ (Step 2)	A Ni(OH)_2_/NiOOH-mediated temporal separation strategy that suppresses carbon loss & catalyst poisoning while boosting energy efficiency	None H^+^ flux from the anode in step 1.	[91]
Flow cell (with GDE)	2H_2_O → H_2_O_2_ + 2H^+^ + 2e^−^	AEM maintains the directional migration of CO_3_^2−^/HCO_3_^−^ to balance the charge and prevent crossover be-tween the electrodes.	Effective anode H^+^ buffering by K_2_CO_3_ aq. for minimal cathode flux impact.	[92]
Flow cell (with GDE)	N_2_H_4_ + 4OH^−^ → N_2_ + 4H_2_O + 4e^−^	CEM maintains the directional migration of K^+^ to balance the charge and prevent crossover be-tween the electrodes.	Negligible H^+^ flux from the anode	[95]
Flow cell (with GDE)	HS^−^ + OH^−^ → S + H_2_O + 2e^−^	CEM maintains the directional migration of K^+^ to balance the charge and prevent crossover between the electrodes.	Negligible H^+^ flux from the anode	[96]

## Data Availability

Data are available in the source publications listed in the bibliography.

## Abbreviations

The following abbreviations are used in this manuscript:

OER	Oxygen Evolution Reaction
CO_2_RR	Carbon Dioxide Reduction Reaction
FE	Faradaic Efficiency
EE	Energy Efficiency
STY	Space–Time Yield
TEA	Techno-Economic Analysis
HER	Hydrogen Evolution Reaction
CEM	Cation Exchange Membrane
AEM	Anion Exchange Membrane
BPM	Bipolar Membrane
GDE	Gas Diffusion Electrode
GDL	Gas Diffusion Layer
MEA	Membrane Electrode Assembly
HAT	Hydrogen Atom Transfer
OOR	Organic Oxidation Reaction
IOR	Inorganic Oxidation Reaction
PCET	Proton-Coupled Electron Transfer
MOR	Methanol Oxidation Reaction
MF	Methyl Formate
FDCA	2,5-Furandicarboxylic Acid
HMF	5-Hydroxymethylfurfural
EO	Ethylene Oxide
IOR	Iodide Oxidation Reaction
DMC	Dimethyl Carbonate
HOR	Hydrogen Oxidation Reaction
HzOR	Hydrazine Oxidation Reaction
SOR	Sulfide Oxidation Reaction
XAS	X-ray Absorption Spectroscopy
TEM	Transmission Electron Microscopy
XRD	X-ray Diffraction
ML	Machine Learning
